# Supplementary material for: Modeling glioblastoma heterogeneity as a dynamic network of cell states
Source: Mol Syst Biol. 2021 Sep 16;17(9):e10105. doi: 10.15252/msb.202010105 (PMC8444284; doi:10.15252/msb.202010105)
Supplement: Supplementary file 6 — Source Data for Figure 5 [file MSB-17-e10105-s004.zip › Figure5A_sourcedata/GSEA_3017/hallmarks_stateD.GseaPreranked.1621934542601/HALLMARK_ADIPOGENESIS.html]

Details for gene set HALLMARK\_ADIPOGENESIS[GSEA]

|  || Dataset | state23017 |
| Phenotype | NoPhenotypeAvailable |
| Upregulated in class | na\_neg |
| GeneSet | HALLMARK\_ADIPOGENESIS |
| Enrichment Score (ES) | -0.32124683 |
| Normalized Enrichment Score (NES) | -1.1581991 |
| Nominal p-value | 0.31009614 |
| FDR q-value | 0.4188852 |
| FWER p-Value | 1.0 |
Table: GSEA Results Summary

  

Fig 1: Enrichment plot: HALLMARK\_ADIPOGENESIS      
 Profile of the Running ES Score & Positions of GeneSet Members on the Rank Ordered List

  

| PROBE | GENE SYMBOL | GENE\_TITLE | RANK IN GENE LIST | RANK METRIC SCORE | RUNNING ES | CORE ENRICHMENT || 1 | SUCLG1 |  |  | 200 | -0.253 | -0.1207 | No |
| 2 | TANK |  |  | 238 | -0.258 | -0.1158 | No |
| 3 | IFNGR1 |  |  | 288 | -0.263 | -0.1195 | No |
| 4 | RMDN3 |  |  | 302 | -0.264 | -0.0954 | No |
| 5 | CHCHD10 |  |  | 430 | -0.277 | -0.1571 | No |
| 6 | ABCA1 |  |  | 563 | -0.293 | -0.2206 | Yes |
| 7 | CMBL |  |  | 609 | -0.303 | -0.2160 | Yes |
| 8 | CCNG2 |  |  | 623 | -0.305 | -0.1866 | Yes |
| 9 | ACADM |  |  | 636 | -0.308 | -0.1561 | Yes |
| 10 | PGM1 |  |  | 715 | -0.322 | -0.1743 | Yes |
| 11 | HADH |  |  | 754 | -0.333 | -0.1605 | Yes |
| 12 | LIFR |  |  | 755 | -0.333 | -0.1175 | Yes |
| 13 | PFKL |  |  | 771 | -0.337 | -0.0855 | Yes |
| 14 | GPHN |  |  | 947 | -0.394 | -0.1688 | Yes |
| 15 | CDKN2C |  |  | 1067 | -0.454 | -0.2015 | Yes |
| 16 | UBC |  |  | 1102 | -0.469 | -0.1670 | Yes |
| 17 | LPL |  |  | 1304 | -1.038 | -0.1871 | Yes |
| 18 | SPARCL1 |  |  | 1314 | -1.537 | 0.0046 | Yes |
Table: GSEA details [plain text format]

  

Fig 2: HALLMARK\_ADIPOGENESIS: Random ES distribution      
 Gene set null distribution of ES for **HALLMARK\_ADIPOGENESIS**
